# Supplementary material for: Synthesis, Cyclooxygenases Inhibition Activities and Interactions with BSA of N-substituted 1H-pyrrolo[3,4-c]pyridine-1,3(2H)-diones Derivatives
Source: Molecules. 2020 Jun 25;25(12):2934. doi: 10.3390/molecules25122934 (PMC7355801; doi:10.3390/molecules25122934)
Supplement: Supplementary file 1 [file molecules-25-02934-s001.pdf]

SUPPLEMENTARY DATA

Synthesis, cyclooxygenases inhibition activities and interactions with BSA of N-substituted 1H-pyrrolo[3,4-c]pyridine-1,3(2H)-diones derivatives

Edward Krzyżak[1]*, Dominika Szkatuła[2], Benita Wiatrak[3], Tomasz Gębarowski[3], Aleksandra Marciniak[1]

1 Department of Inorganic Chemistry, Wroclaw Medical University, uBorowska 211a, 50-556 Wrocław, Poland; edward.krzyzak@umed.wroc.pl; aleksandra.marciniak@umed.wroc.pl

2 Department of Medicinal Chemistry , Wroclaw Medical University, Borowska 211, 50-556 Wroclaw, Poland; dominika.szkatula@umed.wroc.pl

3 Department of Basic Medical Sciences, Wroclaw Medical University, Borowska 211, 50-556 Wroclaw, Poland; benita.wiatrak@umed.wroc.pl; tomasz.gebarowski@umed.wroc.pl

* Correspondence: edward.krzyzak@umed.wroc.pl

NMR spectra

Compound A: - 5,6-dimethyl-4-oxo-2-[(4-phenyl-1-piperazinyl)methyl]-1H-pyrrolo[3.4-c]pyridine-1,3(2H)-dione

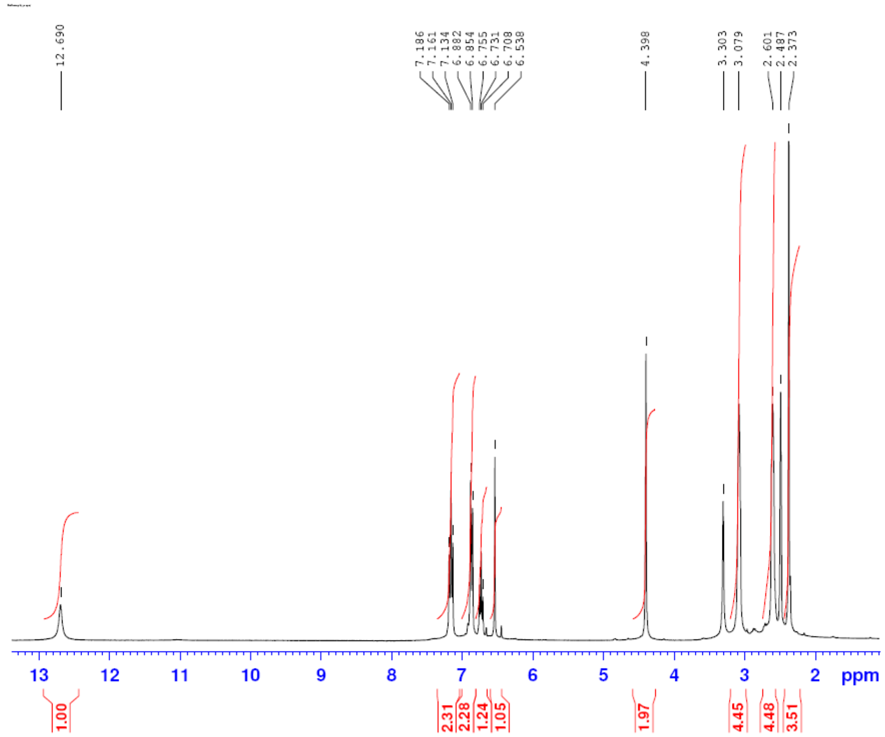

Compound B: - 5,6-dimethyl-4-oxo-2-[4-(3-trifluormethyl)phenyl-1-piperazinyl)methyl]-1H-pyrrolo[3.4-c]pyridine-1,3(2H)-dione

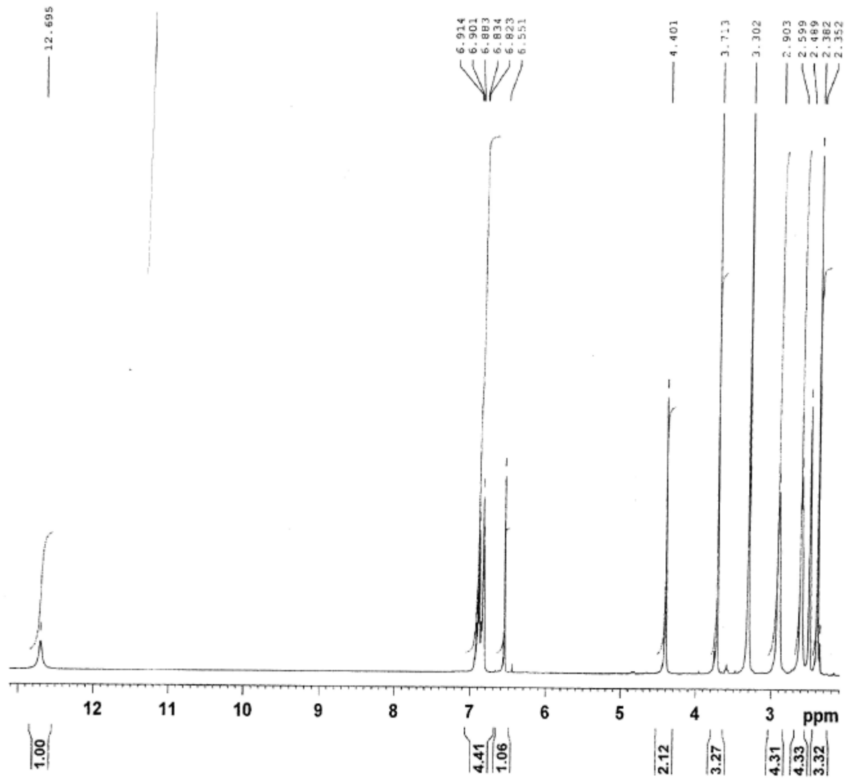

Compound C: - 5,6-dimethyl-4-oxo-2-[4-(2-methoxy)phenyl-1-piperazinyl)methyl]-1H-pyrrolo[3.4-c]pyridine-1,3(2H)-dione

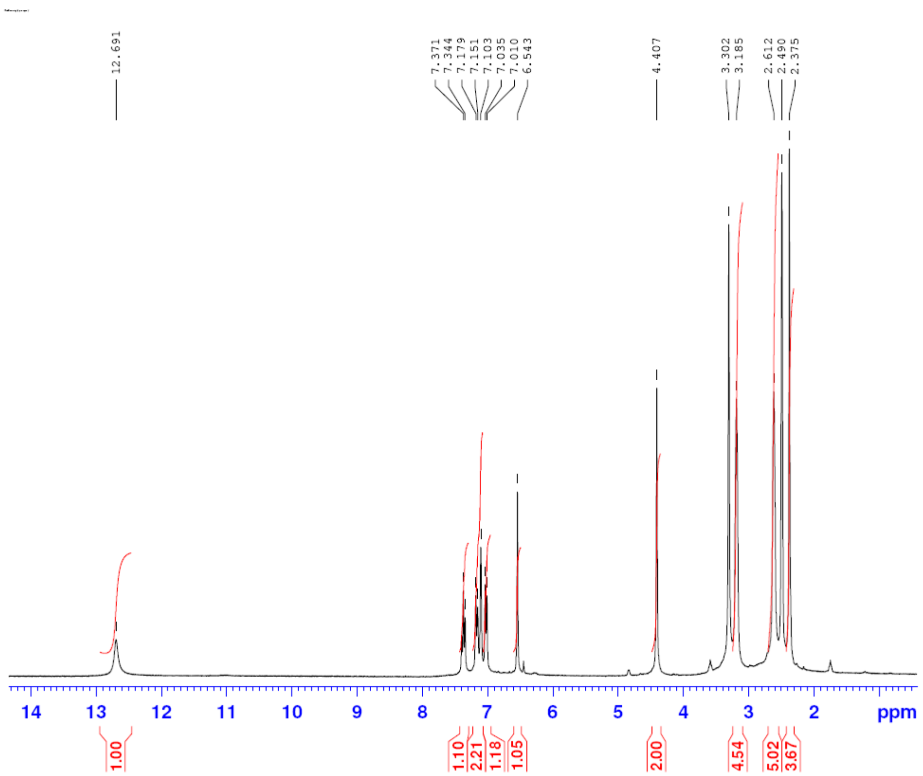

Compound D: - 4-Methoxy–N-[1-(N-pyrrolidine)-methyl]-6-methyl-1H-pyrrolo[3.4-c]pyridine-1,3(2H)-dione

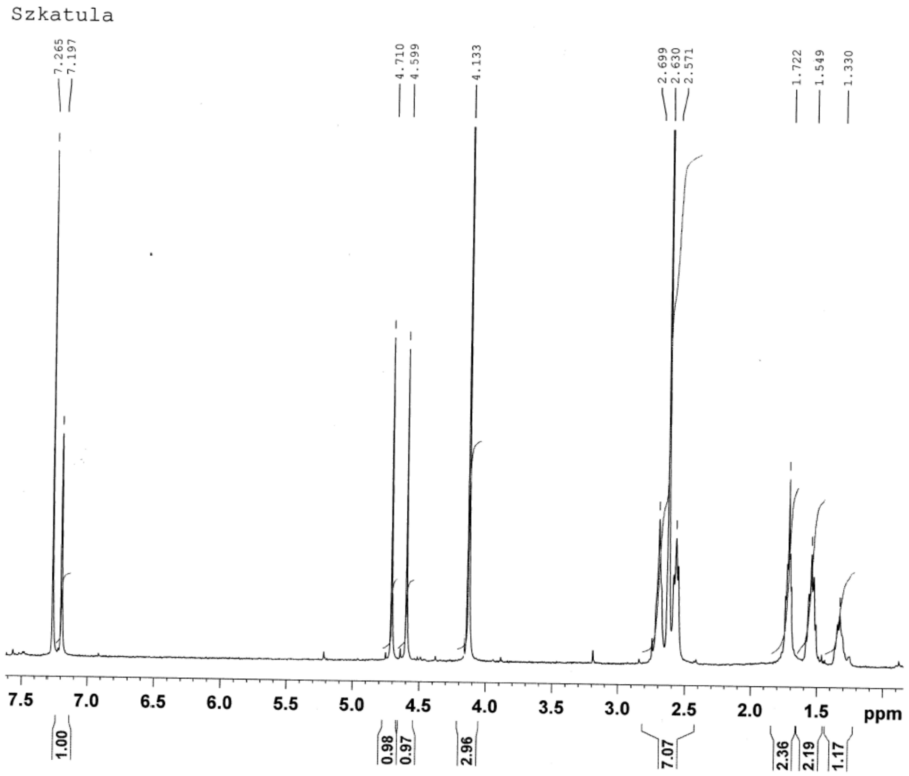

Compound E: - 4-Methoxy–N-[2-(N-morpholine)-ethyl]-6-methyl-1H-[pyrrolo[3.4-c]pyridine-1,3(2H)-dione

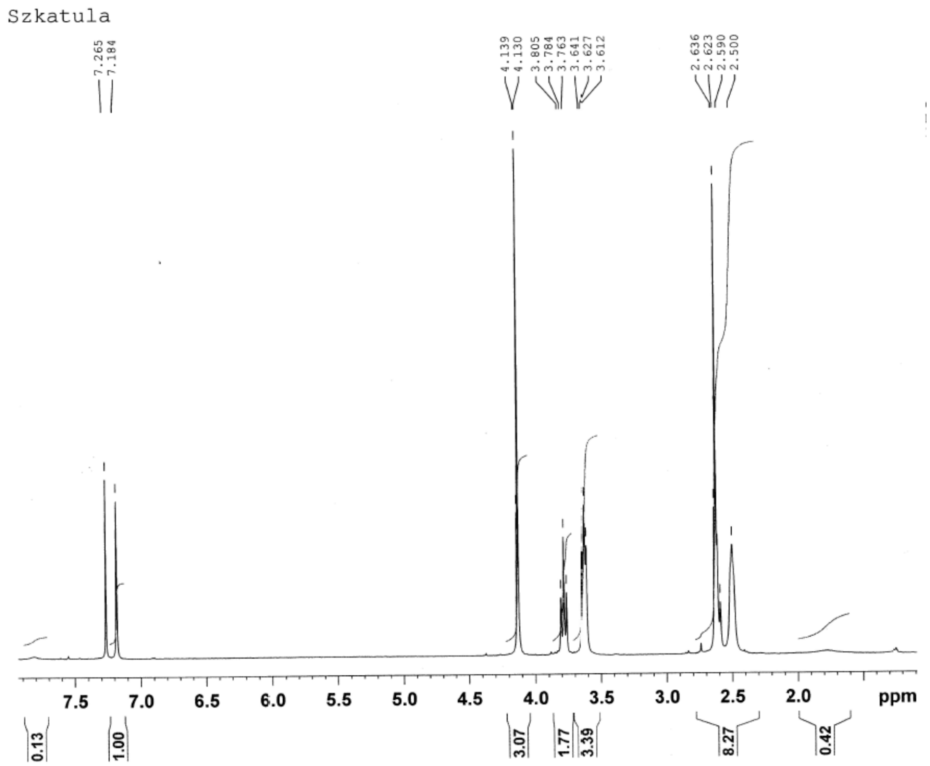

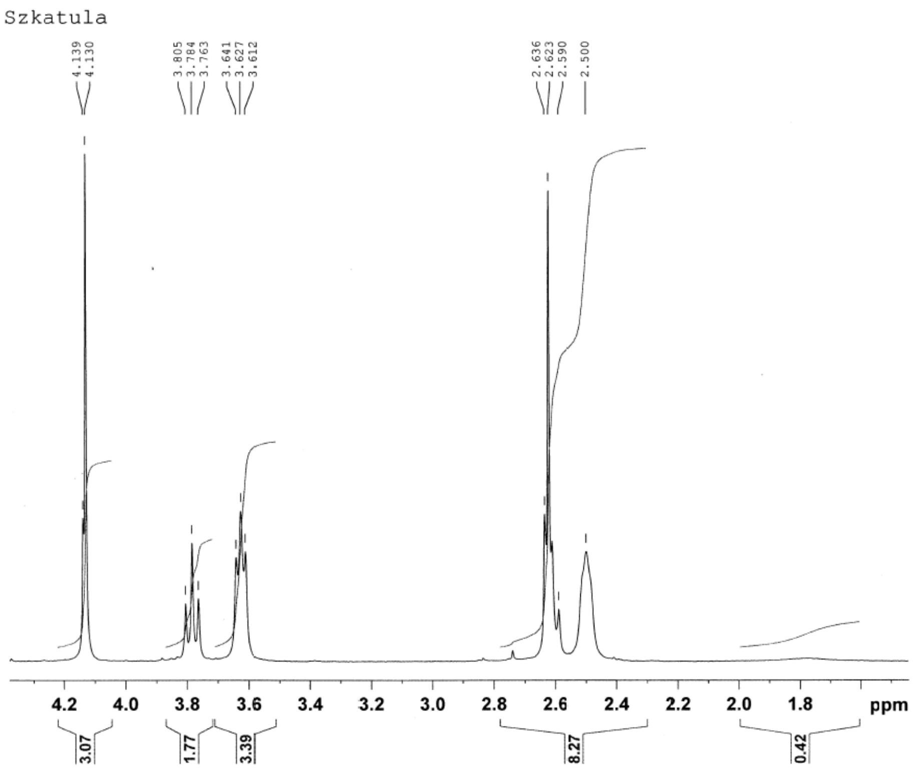
Szkatula
4.139
4.130
3.805
3.784
3.763
3.641
3.627
3.612
2.636
2.623
2.590
2.500
4.2
4.0
3.8
3.6
3.4
3.2
3.0
2.8
2.6
2.4
2.2
2.0
1.8
ppm
3.07
1.77
3.39
8.27
0.42
